# Above- and Below-Ground Carbon Stocks in an Indigenous Tree (*Mytilaria laosensis*) Plantation Chronosequence in Subtropical China

**DOI:** 10.1371/journal.pone.0109730

**Published:** 2014-10-24

**Authors:** Angang Ming, Hongyan Jia, Jinlong Zhao, Yi Tao, Yuanfa Li

**Affiliations:** 1 Experimental Center of Tropical Forestry, Chinese Academy of Forestry, Pingxiang, Guangxi, China PR; 2 Institute of Forest Ecology, Environment and Protection, Chinese Academy of Forestry, Beijing, China PR; 3 Guangxi Youyiguan Forest Ecosystem Research Station, Pingxiang, Guangxi, China PR; 4 College of Forestry, Beijing Forestry University, Beijing, China PR; 5 College of Forestry, Guangxi University, Nanning, Guangxi, China PR; Tennessee State University, United States of America

## Abstract

More than 60% of the total area of tree plantations in China is in subtropical, and over 70% of subtropical plantations consist of pure stands of coniferous species. Because of the poor ecosystem services provided by pure coniferous plantations and the ecological instability of these stands, a movement is under way to promote indigenous broadleaf plantation cultivation as a promising alternative. However, little is known about the carbon (C) stocks in indigenous broadleaf plantations and their dependence on stand age. Thus, we studied above- and below-ground biomass and C stocks in a chronosequence of *Mytilaria laosensis* plantations in subtropical China; stands were 7, 10, 18, 23, 29 and 33 years old. Our assessments included tree, shrub, herb and litter layers. We used plot-level inventories and destructive tree sampling to determine vegetation C stocks. We also measured soil C stocks by analyses of soil profiles to 100 cm depth. C stocks in the tree layer dominated the above-ground ecosystem C pool across the chronosequence. C stocks increased with age from 7 to 29 years and plateaued thereafter due to a reduction in tree growth rates. Minor C stocks were found in the shrub and herb layers of all six plantations and their temporal fluctuations were relatively small. C stocks in the litter and soil layers increased with stand age. Total above-ground ecosystem C also increased with stand age. Most increases in C stocks in below-ground and total ecosystems were attributable to increases in soil C content and tree biomass. Therefore, considerations of C sequestration potential in indigenous broadleaf plantations must take stand age into account.

## Introduction

Biomass and carbon (C) stocks in forest ecosystems play important roles in the global C cycle [1], [2], [3], [4]. Trees and soils are components of forest ecosystems that provide the largest potential for C storage [3], [5], [6], [7], [8], [9]. Increasing global C sequestration through enlargement of the proportion of forested land on the planet has been suggested as an effective measure for mitigating elevated concentrations of atmospheric carbon dioxide [10], [11], [12]. As the area of natural stands has decreased in recent decades, tree plantations have become increasingly important components of the planet's forest resources. Commercial plantations are now a central issue in sustainable forest management across the globe. Well-designed, multi-purpose plantations can reduce pressure on natural forests, restore some ecological services provided by natural forests and mitigate climate change through direct C sequestration [13].

China's large plantation programme is assuming an increasingly significant role in C sequestration from the atmosphere. The total land area under tree plantations has reached 6.2×10^7^ ha and now accounts for 31.8% of the total forested landscape in the country [14]. The largest proportion (63%) of the total plantation area in China is located in subtropical regions, which provide hot and humid conditions appropriate for tree growth [15]. Most of these subtropical plantations consist of stands containing either a single coniferous species or an exotic tree (e.g. *Pinus massoniana*, *Cunninghamia lanceolata*, *Eucalyptus*) [15]. The creation of monospecific stands of trees that are not native to a landscape carries a high risk of consequential ecological damage, such as decrease in ecosystem stability and outbreak of diseases and insect pests [16], [17], [18], [19], [20]. As a result, alternative plantations of indigenous broadleaf species are spreading in this region of China and in neighbouring countries [21], [22], [23], [24], [25], [26], [27].

*Mytilaria laosensis*, an indigenous broadleaf tree species, has potential in the afforestation of subtropical China. It grows rapidly and is strongly adaptable; the trunk is straight and the wood has desirable properties for the economic production of high-value timber. The species occurs naturally in western Guangdong, south-western Guangxi and south-eastern Yunnan. It is also indigenous to Vietnam and Laos. *M. laosensis* is expected to become a major afforestation species in subtropical China and beyond [28], [29], [30]. Its growth patterns, biomass production and wood properties, and the physical and chemical properties of the soils in which it grows have been reported in earlier literature [28], [31], [32], [33]. However, information on biomass and C stocks in *M. laosensis* stands is still lacking. According to previous investigations, C stock size in plantations (especially biomass C) is related not only to tree species, site conditions and soil properties [34], [35], [36], but also to stand age. According to the previous studies, the C stock of *Castanopsis hystrix* plantations and *Erythrophleum fordii* plantation in subtropical China were increased with the increase in stand age [38], [39]. And in the other region, stand age can also remarkably affect the C stocks of plantations' ecosystem [10], [37]. However, Effects of diverse factors, including stand age, on C sequestration by *M. laosensis* plantation are poorly documented [33].

Here, we provide first measurements of the C stock across an age sequence of six *M. laosensis* plantation stands. Specifically, the objectives of this study were (1) to document the changes of the sizes and proportional contributions of plantation C pools as stands aged in the early decades following plantation establishment, and (2) to provide baseline information for forest biomass and C estimations focussing on indigenous broadleaf plantations in subtropical regions.

## Materials and Methods

### Study site and plot establishment

#### Ethics statement

This research was conducted in Experimental Center of Tropical Forestry, Chinese Academy of Forestry (ECTF for short), This study was also supported by this center. We confirmed that the location is not privately owned and the sampling of soils and plants was approved by ECTF. We also confirmed that the field studies did not involve endangered or protected species.

#### Study site

The study site is located in the Experimental Centre of Tropical Forestry at the Chinese Academy of Forestry location in Pingxiang City, Guangxi Zhuang Autonomous Region, China (22°02′–22°19′N, 106°43′–106°52′E). The region is within a semi-humid southern subtropical monsoon climate zone that has defined dry and wet seasons. The dry season extends from October to the following March, and the wet season from April to September. The annual mean precipitation at the site is 1200–1500 mm, annual average evaporation is 1261–1388 mm and the relative humidity is 80–84%. The annual mean temperature is 21°C, with a mean monthly minimum of 12.1°C and a mean monthly maximum of 26.3°C. The landscape is largely comprised of low mountains and hills at elevations of 350–650 m. The soils at the study site are categorised as red soils by the Chinese soil classification procedure; this category is equivalent to Oxisol in the USDA Soil Taxonomy. Most developed from granite and had a sandy texture [40], [41], [42].

#### Plot establishment

In 2013,six adjacent plantations with different stand ages were selected based on similarities in topography, soil texture, management methodology, environmental conditions and previous vegetation composition (dominated by *C. lanceolata*). We identified a chronosequence of *M. laosensis* stands that were 7, 10, 18, 23, 29 and 33 years of age. All six stands were located within 15 km of one another. They were established in 2006, 2003, 1995, 1990, 1984 and 1980, after the clear-cutting of previous *C. lanceolata* vegetation. Stand characteristics are summarised in Table 1.

**Table 1:** Site properties and vegetation characteristics of the six plantation stands studied (values are means ±SE; n = 4).

	7 yr old	10 yr old	18 yr old	23 yr old	29 yr old	33 yr old
Altitude (m)	360–520	450–530	450–540	550–650	450–550	350–500
Slope aspect	North-western	Northern	North-western	Northern	Northern	North-western
Slope gradient (°)	36.1±2.4	37.7±3.1	34.2±1.8	32.2±2.2	32.8±2.6	28.9±1.7
DBH (cm)	13.6±0.7	16.2±1.2	20.2±1.2	23.4±1.7	26.0±2.3	27.3±2.7
Tree height (m)	13.6±1.4	14.9±1.1	16.1±1.7	17.8±2.7	21.9±3.1	20.5±1.9
Stem density (trees ha^−1^)	1500±21	1224±18	911±12	721±11	675±8	671±10
Main understorey species	*M. laosensis*, *Cunninghamia lanceolata*	*M. laosensis*, *Thysanolaena maxima*	*M. laosensi*, *T. maxima*	*M. laosensis*, *C. lanceolata*	*M. laosensis*, *C. lanceolata*	*M. laosensis*, *T. maxima*

During the summer of 2013, four sampling plots (each 30×20 m) were established at random locations in each of the six stands. In each of these plots, we measured the diameter at breast height (DBH, diameter at breast height) of individual trees using a diameter tape, and measured heights using a Hag-löf-VERTEX IV clinometer. Five subplots containing shrubs (each 2×2 m) and five subplots of herbaceous vegetation and litter (each 1×1 m) were established at random locations within each sampling plot (20 subplots in total in the stands of the same age). Plant species, numbers, heights and coverages were recorded; litter was collected from litter subplots (each 1×1 m). Environmental factors including altitude, slope, aspect and slope position were also recorded.

### Measurements

#### Tree biomass

On the basis of the DBH and height measurements in the sampling plots, we selected and harvested six sample trees from different diameter classes in each of the six stands for biomass measurements (36 trees in total). The above-ground portions of the trees were divided into 2-cm sections for measurement. We measured the fresh weights of stems, bark, branches and leaves. The below-ground portions of the sample trees were dug out and examined using the open cut method. We measured the fresh weights of the stump roots, thick roots (diameter >2.0 cm), medium-thick roots (diameter 0.5–2.0 cm) and small roots (diameter <0.5 cm). Organ samples were collected (200 g of each organ) and oven-dried at a temperature of 65°C to constant weight to calculate the moisture contents and dry weights. We built regression models for the different organs to estimate tree biomass (using data from the 18 sample trees).

#### Understorey vegetation and litter biomass

We used a destructive harvesting method to measure the biomass in the above-ground and below-ground portions of the shrub and herbaceous layers [43]. The fresh masses of these two portions were obtained directly by weighing. After oven-drying to constant weight at 65°C, we weighed subsamples and calculated the respective dry weights from determinations of moisture contents. The components of understorey vegetation were then separated and measured. We weighed litter material that had not decomposed or was semi-decomposed at the same time. The litter samples were oven dried at 65°C and weighed.

#### C content

Samples of the above-ground and below-ground components of the sample trees (*M. laosensis*), shrubs, herbaceous plants and litter were dried, ground and sieved in the laboratory. These samples were then bottled for later chemical analysis. In total, 288 soil-sampling points were selected within the 24 sampling plots in each of the six stands. Soil pits were dug to a depth of 100 cm and samples were collected randomly from four depth horizons: 0–10 cm, 10–30 cm, 30–50 cm and 50–100 cm. Soil samples from the same depth horizon in the same stand were mixed in equal proportions and the mixtures were air-dried at room temperature (25°C). The samples were then ground and passed through a 2-mm-mesh sieve to remove coarse living roots and gravel; they were then ground in a mill in preparation for sieving through a 0.25-mm mesh before chemical analysis. A soil-sample cutting ring (100 cm^3^) was used to collect samples of undisturbed soil from different horizons. These samples were taken to the laboratory for measurements of soil bulk density using the cutting ring method. We measured the C content of the tree component samples, understorey vegetation, litter by vario Macro Elemental Analyzer (Elementar Analyasensysteme GmbH, Germany), but the soil organic carbon was established by the oil-bath K_2_Cr_2_O_7_ titration method.

#### C storage

The C stocks (C in biomass per unit area of land surface) in the vegetation and litter biomass were determined by multiplying C content by biomass (dry mass per unit area of land surface). The C stocks per unit area of land in each of the soil horizons were calculated by multiplying soil bulk density at a chosen soil depth by the C content at that depth. Total soil C stocks were computed by summing the stocks in each soil horizon.

### Statistical analysis

We used one-way ANOVA to test for differences in the C content and C stock among plantations of different ages. The dependent parameters were normally distributed and homoscedastic. All analyses were performed using the following software: Microsoft Excel 2007 and SPSS (ver.13.0; SPSS, Chicago, IL) for Windows. Statistical significance was detected at *P*<0.05.

## Results

### C content in plantation stands

#### C content in the vegetation and litter layer

The C contents of the component organs differed significantly among the six stands (*P*<0.05) and fell into the following rank order: leaf > stem > coarse root > medial root > bark > small root > branch > stump root > fruit. C content was not significantly different between medium roots and bark (*P*<0.05). The C contents of above-ground components of the shrub and herb layers in all six stands were higher than those of their below-ground components. In the litter layer, the C content of the undecomposed portion was higher than that of the semi-decomposed portion (Table 2).

**Table 2:** Carbon contents in the vegetation components and litter layers of six differently aged plantation stands (values are means ±SE; n = 4 g kg^−1^).

Layer	Components	7 yr old	10 yr old	18 yr old	23 yr old	29 yr old	33 yr old	Mean
Tree layer	Stem	527.8±14.1^Eb^	536.9±12.7^Cb^	545.6±16.4^Bab^	520.7±29.3^Cb^	556.7±15.2^Ba^	569.1±34.7^ABa^	542.8±20.4^Cb^
	Bark	524.4±11.6^Fa^	534.8±31.2^Ca^	554.1 ±24.8^ABa^	506.8±13.7^Da^	543.2±19.4^Ca^	517.9±18.8^Da^	530.2±22.3^Da^
	Branches	534.1±27.6^Da^	512.5±21.8^Ea^	532.2±31.5^Ca^	498.8±33.2^Eb^	516.9±19.7^Ea^	542.3 ±41.2^Ba^	522.8±28.6^Ea^
	Leaves	573.6±44.7^Aa^	525.9 ±31.2^Ca^	547.1±28.8^Ba^	560.2±42.9^Aa^	568.3±44.4^Aa^	586.1±36.1^Aa^	560.2±38.4^Aa^
	Fruit	512.4±17.2^Ha^	498.8 ±21.8^Fa^	505.2±15.4^Ea^	489.7±17.7^Fa^	504.3±21.9^Fa^	488.4±20.4^Ea^	499.8±18.8^Ha^
	Stump roots	514.3±22.2^Ga^	533.7±27.4^Ca^	498.2±23.7^Fb^	509.6±14.9^Dab^	532.1±20.8^Da^	508.1±24.6^DEa^	516.0±23.8^Fa^
	Coarse roots	543.4±31.8^Ca^	554.1±37.1^Aa^	527.6±30.4^Ca^	538.7±28.6^Bab^	520.8±27.3^Eb^	558.4±40.2^Ba^	540.5±33.6^Cab^
	Medium roots	517.9±30.2^Fa^	541.7±37.7^Ba^	532.8±24.9^Ca^	557.4±40.2^Aa^	531.0±23.4^Da^	520.2±28.7^Ca^	533.5±32.6^Da^
	Small roots	544.1±23.8^Ca^	537.0 ±31.9^BCa^	515.6±21.3^Da^	520.4±19.4^Ca^	536.3±33.1^CDa^	525.4±29.2^Ca^	529.8±25.2^Da^
	Average of roots	529.9±26.8^Ea^	541.6±34.2^Ba^	518.6±21.7^Da^	531.5±23.7^BCa^	530.1±27.4^Da^	533.7±28.9^Ca^	530.9±28.3^Da^
	Average of trees	538.0±25.5^Da^	530.3±30.4^Ca^	539.5±27.1^BCa^	523.6±34.2^Ca^	543.0±27.3^Ca^	549.9 ±26.5^Ba^	537.4±29.7^CDa^
Shrub layer	Above ground	544.2±32.2^Ca^	516.7±21.7^Da^	545.2±31.8^Ba^	534.8±25.5^Ba^	541.7±27.4^Ca^	522.2±18.9^Ca^	534.1±25.8^Da^
	Below ground	522.3±17.3^Fa^	522.4 ±19.1^CDa^	497.5±20.4^Fa^	508.4±24.3^Da^	516.8±19.4^Ea^	519.2±9.7^CDa^	514.4±18.3^FGa^
	Average	533.3±28.3^Da^	519.6±19.6^Da^	521.4±25.3^Ca^	521.6±14.8^Ca^	529.3±25.4^Da^	520.7±13.7^Ca^	524.3±22.9^DEa^
Herb layer	Above ground	513.3±31.7^Ha^	532.8±28.8^Ca^	527.9±35.4^Ca^	508.4±31.0^Da^	529.1±19.8^Da^	511.7±27.6^Da^	520.5±30.7^Ea^
	Below ground	518.4±27.2^Fa^	509.3±19.7^Ea^	497.6±16.9^Fa^	509.8±21.3^Da^	524.2±24.7^DEa^	510.5±18.8^Da^	511.6±20.6^Ga^
	Average	515.9±29.7^Ga^	521.05 ±25.4^Da^	512.8±27.3^Ea^	509.1±26.8^Da^	526.7±22.3^Da^	511.1±25.2^Da^	516.1±26.4^Fa^
Litter layer	Undecomposed	557.8±32.2^Ba^	578.4±40.4^Aa^	562.1±34.2^Aa^	550.9±27.9^ABa^	564.2±40.1^Aa^	578.5±31.2^Aa^	565.3±35.7^A^
	Semi-decomposed	554.2±29.9^Ba^	543.8±31.0^Ba^	542.2±27.6^Ba^	537.1±30.4^Ba^	529.7±27.7^Da^	547.0±29.4^Ba^	542.3±28.3^C^
	Average	556.0±31.3^Ba^	561.1±36.4^Aa^	552.2±30.9^Ba^	544.0±28.7^Ba^	547.0±35.1^Ca^	562.8±30.1^Ba^	553.8±31.4^B^

Note: Different Capital letters in the same list indicate significant pairwise differences within stand ages between components, and different lowercases indicate significant pairwise differences within components between stand ages (multiple comparisons test; *P*<0.05).

The average C content did not differ significantly within plantation components among stands of different ages (*P*>0.05). No obvious pattern relationships were detected between C contents and increasing stand age.

#### C content in the soil layer

The C content changed markedly with increasing soil depth in all six stands (*P*<0.01; Table 3). The value in the topsoil (0–10 cm depth) was 46.1% higher than the average at 100 cm depth.

**Table 3:** Carbon contents by soil depth in six differently aged *Mytilaria laosensis* plantation stands (values are means ±SE, n = 12; g kg^−1^).

Soil depth (cm)	7 yr old	10 yr old	18 yr old	23 yr old	29 yr old	33 yr old	Mean
0–10	28.1±4.2^A^	27.4±2.8^A^	29.6±2.9 ^A^	31.7±2.4^AB^	39.7±4.3^C^	47.1±5.7^D^	33.9±3.4
10–30	23.2±2.7^A^	24.7±2.1^A^	26.3±1.7^B^	26.6±2.3^B^	27.7±1.8^B^	31.0±2.5^C^	26.6±2.4
30–50	18.9±2.4^A^	19.4±2.7^A^	18.6±1.9^A^	19.0±2.6^A^	19.4±3.1^A^	20.5±3.4^A^	19.3±3.0
50–100	10.9±1.5^A^	11.3±1.7^A^	13.2±2.3^B^	12.8±2.0^B^	14.4±2.4^C^	15.4±2.7^C^	13.0±2.3
Mean	20.3±2.5	20.7±2.3	21.9±2.7	22.5±2.4	25.3±3.2	28.5±3.7	23.2±2.7

Note: Different capital letters indicate significant pairwise differences within soil depths between stand ages (multiple comparisons test; *P*<0.05).

The C contents of the two shallowest soil layers (0–10 cm and 10–30 cm), the horizon at 50–100 cm and values obtained by summing across all soil layers increased significantly with increasing stand age (*P*<0.05). The C content in the 3050 cm soil layer, however, did not differ significantly among different ages (*P*>0.05).

### C stocks in plantation stands

#### Biomass and C stocks in tree layers

The allometric relationship between the biomass of the tree organs (*W*) and DBH (*D*) andheight (*H*) were best-fitted with equations in the form *W* = *a*(*D* ^2^ *H*)^b^. The *F*-tests showed that all regressions were highly significant (*P*<0.01). Biomass calculations based on these allometric equations were used in the estimations of C stocks detailed in Table 4.

**Table 4:** Individual biomass regressions models for *Mytilaria laosensis* trees (*n* = 18 for all models); *W*, biomass; *D*, DBH; *H*, height.

Organ	Allometric equation	*R* ^2^	*F*-value	*P*
Stem	*W*_s_ = 0.1740(*D* ^2^ *H*)^0.7661^	0.9196	104.3812	<0.0001
Bark	*W_ba_* = 0.0220(*D^2^H*)^0.7081^	0.7191	58.6375	<0.0001
Branches	*W_br_* = 0.0002(*D* ^2^ *H*)^1.2696^	0.6291	11.2124	0.0065
Leaves	*W_l_* = 0.00003(*D*)^1.2634^	0.8091	41.0306	0.0001
Roots	*W_r_* = 0.0094(*D* ^2^ *H*)^0.9538^	0.7247	14.7872	0.0027
Total tree	*W_t_* = 0.1536(*D* ^2^ *H*)^0.8268^	0.9049	64.8073	<0.0001

**Figure 1:**
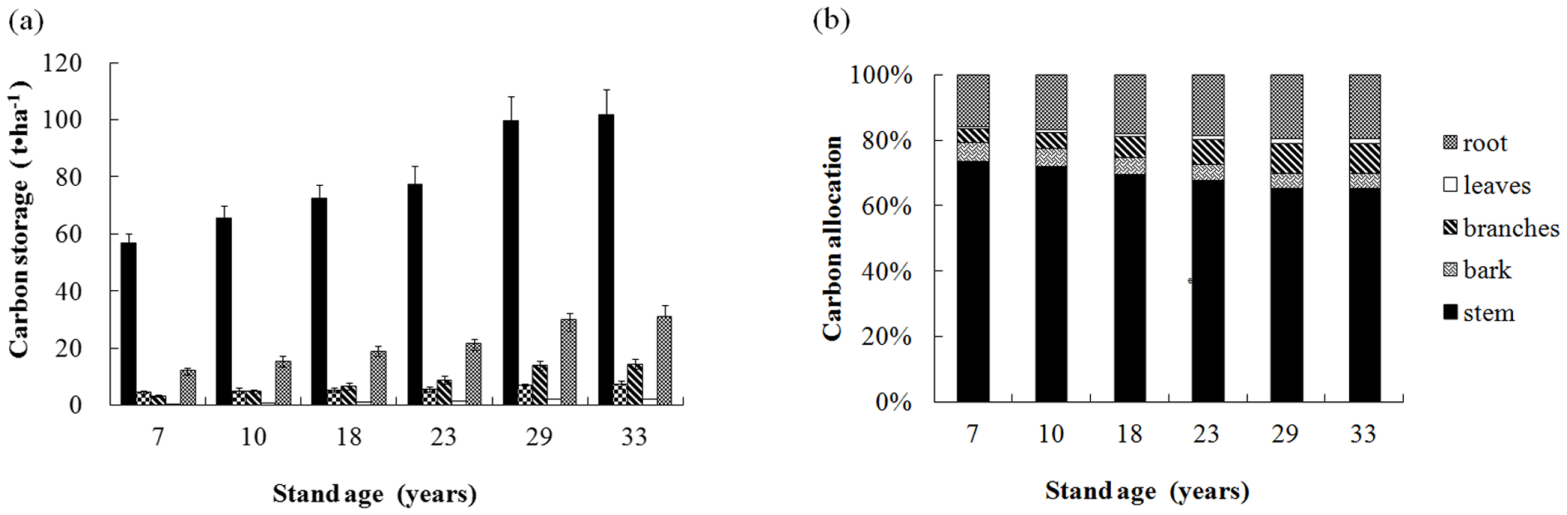
Carbon stocks and their allocation to tree components in six differently aged *Mytilaria laosensis* stands. Values in (a) are means ±SE, n = 4. Note: Different lowercase letters above the bars indicate significant differences between means (multiple comparisons test; *P*<0.05).

Fig. 1a depicts C stocks in stands of different ages and their allocation among component organs. The total C stocks in the trees were 77.3, 91.3, 104.1, 114.6, 153.0 and 156.2 t ha^−1^ in 7-, 10-, 18-, 23-, 29- and 33-year-old stands, respectively. Thus, stocks rapidly increased with age from 7 to 29 years, but plateaued thereafter. C stocks in stems made up 74.0, 72.1, 69.7, 67.7, 65.3 and 65.2% of the total tree content in 7-, 10-, 18-, 23-, 29- and 33-year-old stands, respectively. Furthermore, trends in the C stocks of stems, roots, bark, branches and leaves tracked those of total tree C stocks across all stand ages.

The allocation of C stocks to stems and bark decreased with stand age from 7 to 29 years; stem and bark allocations were similar in 29- and 33-year-old stands. In contrast, C allocations to branches, leaves and roots increased with stand age from 7 to 29 years, but were similar in 29- and 33-year-old stands.

#### C stocks in the shrub, herb and litter layers

**Figure 2:**
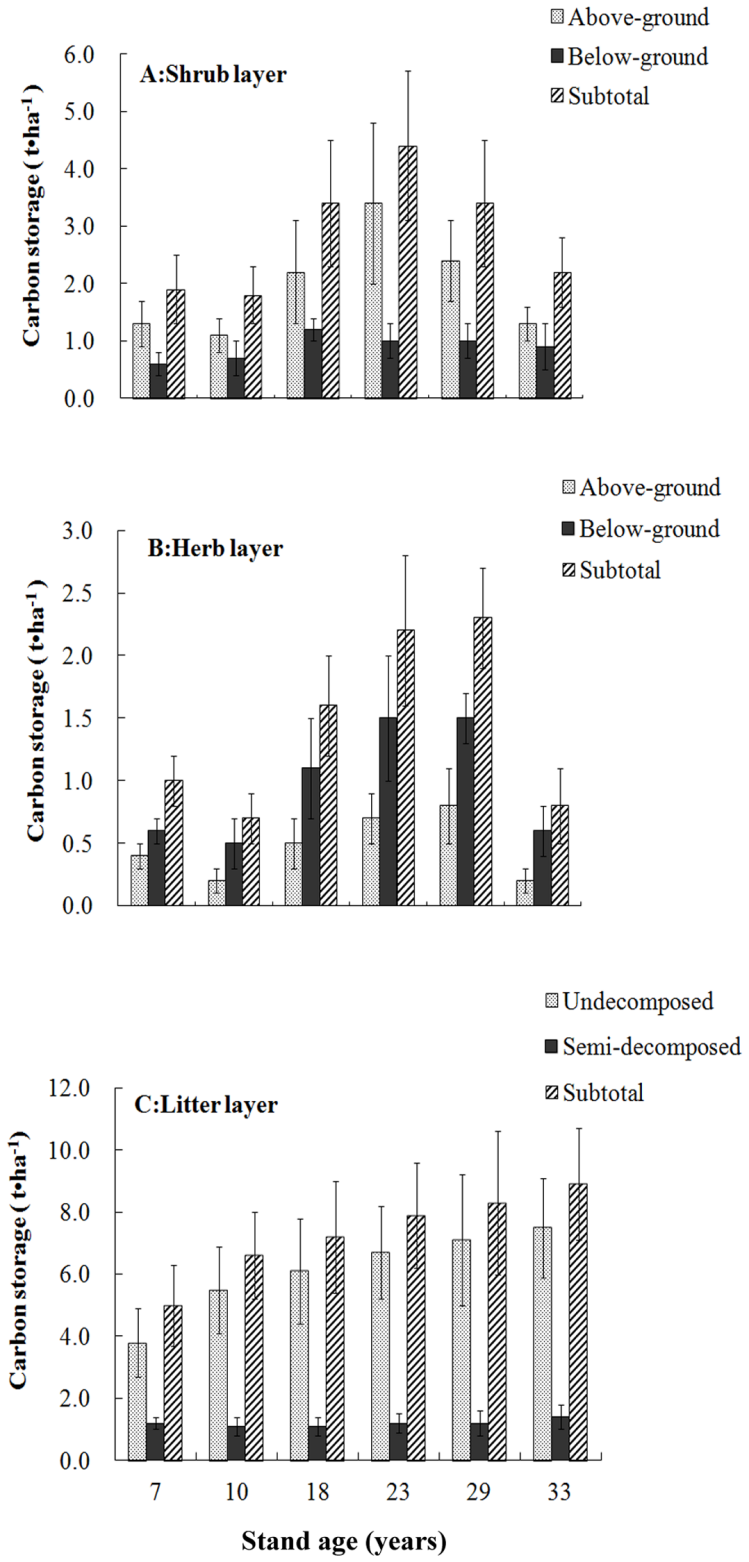
Carbon stocks in the shrub, herb and litter layers in six differently aged *Mytilaria laosensis* stands. Values are means ±SE, n = 4.

The C stock levels of each layer of the six plantation stands are shown in Fig. 2. Small proportions of biomass and C were measured in the shrub, herb and litter layers. The summed C contents of the shrub, herb and litter layers made up 2.9%, 3.0%, 3.6%, 4.0%, 3.2% and 2.6% of the total C in 7-, 10-, 18-, 23-, 29- and 33-year-old stands, respectively. The above-ground C stocks in the shrub layers of the six stands were higher than below-ground shrub stocks. However, above-ground biomass and C stocks in the herb layer were lower than those below ground. The undecomposed biomass and C stocks in the litter layer were higher than those in the semi-decomposed portion; the highest average C stock in the undecomposed litter was 5.1-fold higher than that in the semi-decomposed portion.

Forest ground vegetation C stocks were correlated with stand age across the entire plantation chronosequence. The shrub C stocks increased with stand age from 7 to 23 years (*P*<0.05), but decreased with stand age from 23 to 33 years (*P<0.05*). Herb C stocks increased with stand age from 7 to 29 years, but decreased thereafter. C stocks in semi-decomposed portions of the litter layer were not related to stand age, but those in undecomposed portions and in the combined litter layer increased remarkably with increasing stand age (*P<0.05*). We therefore predict that the C stock in the litter layer will increase continually as the stands become older.

#### C stock in the soil layer

**Figure 3:**
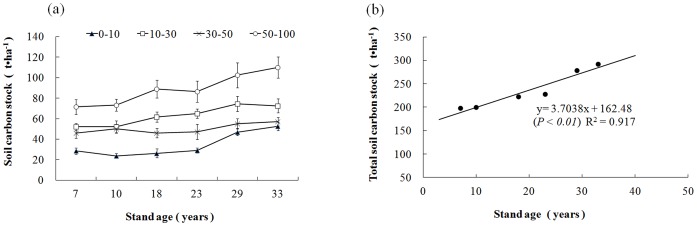
Soil layer carbon stocks in six differently aged *Mytilaria laosensis* stands (a) and the linear relationship between soil carbon stocks and stand age (b). Values are means ±SE n = 12. Soil depth ranges in the key to (a) are in cm units.

C stocks decreased with increasing soil depth even though the soil bulk density increased with depth. Fig. 3 depicts trends in the soil layer C stock across the *M. laosensis* stand age sequence. The top soil (0–10 cm) and deeper soil (50–100 cm) stocks followed an increasing trend with stand age (*P*<0.05), especially in the older stands (the difference between 29- and 33-year-old stands was especially significant; *P*<0.01). Although soil C stocks in the 10–30-cm and 30–50-cm horizons increased with age, the relationship was not significant (Fig. 3a).

Summed C stocks from 0 to 100 cm soil depth were 198.2, 199.6, 222.8, 227.9, 278.7 and 292.0 t ha^−1^ in the 7-, 10-, 18-, 23-, 29- and 33-year-old stands, respectively. This linear trend was significant (Fig. 3b).

#### C stock in the plantation ecosystem

**Figure 4:**
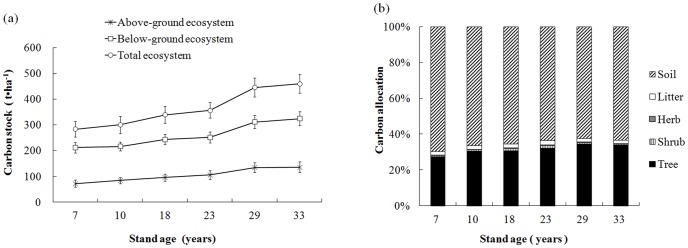
Carbon stocks in ecosystem components (a) and their proportional allocation (b) in six differently aged *Mytilaria laosensis* stands. Values in (a) are means ±SE, n = 4.

Table 5 summarises individual ecosystem C stocks measured within each of the six stands. The rank order of C stock proportions across the six stands was as follows: soil layer (62.6–70.0%) > tree layer (27.3–33.9%) > litter layer (1.8–2.2%) > shrub layer (0.5–1.3%) > herb layer (0.2–0.6). Averaging across stands, 65.3% of the total C was in the soil and 31.5% in the trees (Fig. 4b).

**Table 5:** Carbon stocks and their allocation in six differently aged *Mytilaria laosensis* stands (values are means ±SE, n = 4; t ha^−1^).

Layers	Components	7 yr old	10 yr old	18 yr old	23 yr old	29 yr old	33 yr old	
tree layer	stem	56.8±3.4^F^	65.5±4.2^E^	72.3±4.7^D^	77.4±6.3^C^	99.8±8.5^B^	101.6±9.1^A^	
	bark	4.5±0.5^D^	5.0±1.0^C^	5.4±0.6^B^	5.6±0.8^B^	7.1±0.4^A^	7.2±1.3^A^	
	branches	3.2±0.4^E^	4.7±0.7^D^	6.7±1.3^C^	8.7±1.4^B^	13.9±1.7^AB^	14.4±1.7^A^	
	leaves	0.5±0.2^E^	0.7±0.1^D^	1.0±0.1^C^	1.3±0.1^B^	2.1±0.2^AB^	2.2±0.3^A^	
	total above-ground tree	65.0±2.9^E^	75.9±4.5^D^	85.4±4.9^C^	93.0±5.8^B^	122.9±9.2^AB^	125. 4±10.4^A^	
	root	12.3±0.9^E^	15.4±1.7^D^	18.7±2.1^C^	21.6±1.4^B^	30.1±2.3^AB^	30.8±4.2^A^	
	subtotal	77.3±4.4^E^	91.3±4.7^D^	104.1±5.1^C^	114.6±6.2^B^	153.0±8.4^AB^	156.2±10.3^A^	
shrub layer	above-ground	1.3±0.4^C^	1.1±0.3^D^	2.2±0.9^B^	3.4±1.4^A^	2.4±0.7 ^B^	1.3±0.3^C^	
	below-ground	0.6±0.2^D^	0.7±0.3^C^	1.2±0.2^A^	1.0±0.3^AB^	1.0±0.3 ^AB^	0.9±0.4^B^	
	subtotal	1.9±0.6^D^	1.8±0.5^D^	3.4±1.1^B^	4.4±1.3^A^	3.4±1.1^B^	2.2±0.6^C^	
herb layer	above-ground	0.4±0.1^B^	0.2±0.1^C^	0.5±0.4^B^	0.7±0.2^A^	0.8±0.3^A^	0.2±0.1^C^	
	below-ground	0.6±0.1^C^	0.5±0.2^C^	1.1±0.4^B^	1.5±0.5^A^	1.5±0.2^A^	0.6±0.2^C^	
	subtotal	1.0±0.2^C^	0.7±0.2^D^	1.6±0.4^B^	2.2±0.6^A^	2.3±0.4^A^	0.8±0.3^D^	
litter layer	undecomposed	3.8±0.1.1^D^	5.5±1.4^C^	6.1±1.7^B^	6.7±1.5^B^	7.1±2.1^A^	7.5±1.6^A^	
	semi-decomposed	1.2±0.2^B^	1.1±0.3^B^	1.1±0.3^B^	1.2±0.3^B^	1.2±0.4^B^	1.4±0.4^A^	
	subtotal	5.0±1.3^E^	6.6±1.4^D^	7.2±1.8^C^	7.9±1.7^B^	8.3±2.3^AB^	8.9±1.8^A^	
soil layer	0–10	28.5±3.2^E^	23.9±2.4^F^	26.3±4.2^D^	29.1±2.5^C^	46.9±3.4^B^	52.6±4.1^A^	
	10–30	52.2±3.4^D^	52.1±5.8^D^	61.7±4.8^C^	65.1±4.6^B^	74.3±7.4^A^	72.5±6.7^A^	
	30–50	46.1±5.1^C^	50.3±4.4^B^	45.9±4.7^C^	47.4±7.4^BC^	55.2±4.6^A^	57.0±4.1^A^	
	50–100	71.4±7.4^E^	73.3±5.7^E^	88.8±8.6^D^	86.3±10.4^C^	102.3±12.3^B^	109.9±10.3^A^	
	subtotal	198.2±12.4^E^	199.6±10.3^E^	222.7±11.4^D^	227.9±13.2^C^	278.7±17.3^B^	292.0±16.4^A^	
	above-ground ecosystem	71.7±14.2^E^	83.8±12.4^D^	95.3±14.2^C^	105.0±17.6^B^	134.4±18.8^A^	135.8±20.4^A^	
	below-ground ecosystem	211.7±20.4^F^	216.2±17.8^E^	243.7±19.4^D^	252.0±21.3^C^	311.3±25.6^B^	324.3±27.3^A^	
	total ecosystem	283.4±30.4^F^	300.0±34.1^E^	339.0±33.7^D^	357.0±29.8^C^	445.7±37.1^B^	460.1±36.4^A^	

Note: Different Capital letters in the same row indicate significant pairwise differences within components between stand ages; (multiple comparisons test; *P*<0.05).

Fig. 4a shows changes in C stocks in above-ground, below-ground and total ecosystem components with increasing stand age. Above-ground ecosystem C stocks increased as stand age increased from 7 to 29 years; thereafter, the C content changed little. Below-ground and total ecosystem C stocks increased across the entire age range.

The above-ground to below-ground ecosystem C stock ratios were 0.338, 0.388, 0.391, 0.417, 0.431 and 0.419 in the 7-, 10-, 18-, 23-, 29- and 33-year-old stands, respectively; the ratios increased gradually with age due to the accumulation of above-ground C in tree biomass.

## Discussion

### C content

C content in forest ecosystems varies by forest type. Tree species and site conditions are related to the C content [35], [43]. Across six *M. laosensis* stands with different ages, we detected a significant difference in mean C contents among different tree organs. No significant effect of stand age was detected within individual tree organs in the *M. laosensis* plantations, which corroborates the findings of studies on other species [38], [45].

The C contents of the various vegetation layers in the same stand fell into the following rank order: trees > shrubs > herbaceous plants (Table 2). Trees are probably high ranking because they can synthesise and accumulate more organic matter than can other types of vegetation [46].

The C content of litter varies with many factors, such as tree species, litter productivity, decomposition rate and microenvironment [35], [44]. Litter reportedly decomposes at a considerably faster rate in broadleaf forests than in coniferous stands; thus, the standing stock of C is lower in broadleaf forests [35]. We measured a mean litter C content of 553.8 g kg^−1^ in *M. laosensis* stands. This value is considerably higher than that in *P. massoniana* stands (505.9 g kg^−1^) in subtropical China [43], probably because the leaves of *M. laosensis*, which were the main component of the litter layer, are very leathery and refractory [47].

The mean C content of the soil layers in each stand decreased as the depth increased. The C content in the topsoil was higher than that in the deeper soil because the organic C produced from the decomposition of litter and root systems near the ground surface had entered the topsoil first, as demonstrated in other studies [10], [48], [49]. The C contents of the top two soil layers (0–10 cm and 10–30 cm), the deepest layer (50–100 cm) and across all soil layers significantly increased with increasing stand age, probably due to the increasing litter productivity in older stands. This finding helps to explain the increase in below-ground C stocks as stands age.

### C stocks in plantation stands

Estimating the C stock pools stored in age-sequenced plantations may contribute to forest management for C sequestration. C stocks in trees depend on stand density, biomass and relative C contents in the tissues. We found that C contents were positively correlated with C stocks (Table 5). The tree C stocks increased rapidly with age from 7 to 29 years, but plateaued thereafter, perhaps due to declining tree growth rates. Most previous studies have reported increases throughout the growth phases of trees [10], [38]. The laws of growth may vary among tree species, For example, *Euclyptus urophylla* ×*E.grandis* grew faster in its early years, but turned slowly later, On the contrary, Castanopsis hystrix grew faster in the later stage than that in the early years [35], [38], [39], [45], that is to say, different tree species show different growth charactersAs to *Mytilaria laosensis*, itneeds 25–30 years to reach the largest yield stage [28]. This variation may account for the difference between *Mytilaria laosensis* plantations and most other stands.

Only small portions of biomass and C were sequestered in the shrub, herb and litter layers, which accounted for only 2.6–4.0% of the total. We detected a correlation between forest ground vegetation C stock and stand age across the entire chronosequence. C stocks in ground vegetation increased in the early stages of tree stand development, but decreased in older stands in concert with changes in tree canopy cover and stand density. Nevertheless, no obvious common patterns have been detected in studies on ground vegetation C storage. In the litter layer, the undecomposed and the total litter C stock decreased markedly with increasing stand age, which probably accounts for increases in soil C content with increasing stand age.

Soil was the largest C pool in the six stands that we studied. Soil C stock depends on stand age, the physical and chemical properties of the soil, forest type, litter productivity and litter decomposition rate [6], [35], [43], [50]. We found a significant linear relationship between total soil C stocks across the 0–100-cm-depth range and stand age. Hence, the proportion of C stock below ground will probably increase over protracted periods in the future life of the plantation ecosystems we studied.

Proportions of plantation ecosystem C stocks in the six stands fell into the following rank order: soil layer > tree layer > litter layer > shrub layer > herb layer. Soil and tree biomass harboured the largest C pools in the ecosystems, accounting for 96.8% of the total. These findings are congruent with previous studies [51], [52], [53], [54].

Above-ground ecosystem C stocks increased during the early stages of stand development and plateaued after 29 years due to a deceleration in tree growth. However, below-ground and total ecosystem C stocks increased with the stand age during the whole chronosequence we studied.

## Conclusions

Stand age is a major determinant of C stocks in plantations. Both the C stocks and their distributions among plantation ecosystem components were affected by stand age. We found no significant differences in C contents in above-ground components among stand ages, but the soil C content increased with increasing stand age. Tree C was the largest above-ground ecosystem fraction, which contributed 25.7% to the total ecosystem C stocks in all six stands. The soil fraction was the largest C pool across plantations. C stocks across the 0–100 cm soil depth range increased across the entire chronosequence. They were significantly linearly related to tree growth. The increase in above-ground tree biomass with increasing stand age significantly affected the above-ground ecosystem C stock size. The increases in below-ground ecosystem C stocks through a 7-year to 33-year stand chronosequence were mainly attributable to increases in soil organic C. Thus, one must take into account the successional development in forest ecosystem C pools when estimating C sink potentials over the complete life cycle of plantation stands.
